# Predictive analytics for supply chain resilience in urban infrastructure networks using graph convolutional networks

**DOI:** 10.1371/journal.pone.0345444

**Published:** 2026-08-14

**Authors:** Yanfang Liu, Ruijun Hu, Wen Zhang, Qian Wu

**Affiliations:** 1 School of Management, Beijing University of Chinese Medicine Dongfang College, Cangzhou, Hebei, China; 2 School of Business Administration, Joongbu University, Goyang–si, Republic of Korea; 3 School of Architectural Engineering, Heze Vocational College, Heze, Shandong, China; Shanghai University, CHINA

## Abstract

Urban infrastructure networks underpin modern supply chain operations, yet their vulnerability to disruptions poses significant challenges to urban resilience; understanding and predicting such disruptions requires analytical frameworks that jointly leverage network topology and flow dynamics. This study proposes a three–stage framework integrating complex network analysis, Graph Convolutional Network (GCN)–based predictive modeling, and resilience stress testing, validated on a large–scale New York City dataset comprising 6,302 infrastructure nodes, 96,641 disruption events, and 100,000 traffic flow records. The proposed GCN model achieves an RMSE of 5.8 and R^2^ of 0.92, significantly outperforming ARIMA, SVR, LSTM, and XGBoost baselines, with ablation experiments confirming that graph structure reduces prediction error by 29.3%. Resilience analysis reveals that targeted hub–node attacks cause rapid initial fragmentation, while a crossover effect at approximately 30% node removal shows that random failures induce greater cumulative damage. Temporal factors and historical flow patterns are identified as the dominant predictors, collectively accounting for 65.5% of feature importance. The integrated framework bridges flow prediction and resilience assessment, enabling identification of dual–risk nodes for prioritized infrastructure protection and providing actionable insights for enhancing urban supply chain resilience.

## Introduction

Urban infrastructure networks, encompassing transportation, logistics, and distribution systems, serve as the critical arteries of modern supply chains [1]. These interconnected systems facilitate the movement of goods, services, and information across metropolitan areas, supporting economic activities and daily life [2]. However, the increasing complexity and interdependency of these networks have made them susceptible to various disruptions, ranging from traffic accidents and equipment failures to natural disasters and deliberate attacks.

The concept of supply chain resilience has gained significant attention in recent years, particularly following global disruptions such as the COVID–19 pandemic, which exposed the fragility of interconnected supply networks [3]. Resilience, defined as the ability of a system to anticipate, prepare for, respond to, and adapt to incremental change and sudden disruptions (National Infrastructure Advisory Council, 2009) [4], has become a paramount concern for urban planners, logistics managers, and policymakers.

Predictive analytics offers a promising approach to enhance supply chain resilience by enabling proactive identification of potential disruptions and their cascading effects on network performance [5]. By leveraging historical data on traffic patterns, incident occurrences, and network topology, machine learning models can forecast flow disruptions, assess vulnerability hotspots, and inform strategic planning decisions.

Despite growing interest in this area, significant research gaps remain. First, most existing studies focus on either network topology analysis or predictive modeling in isolation, without integrating these complementary approaches into a unified analytical framework [6]. Second, there is limited empirical research utilizing large–scale, real–world urban infrastructure data to validate theoretical frameworks, with most studies relying on simulated or small–scale datasets [7]. Third, the comparative performance of emerging deep learning methods, particularly graph neural networks that can explicitly leverage network topology, against traditional forecasting techniques in supply chain resilience contexts remains underexplored [8]. Fourth, the relationship between network structural properties and flow prediction performance has not been systematically investigated, leaving a gap in understanding how topological features contribute to predictive accuracy [9].

To address these gaps, this study makes the following contributions: we propose a Graph Convolutional Network (GCN) architecture specifically designed for supply chain flow prediction in urban infrastructure networks that explicitly encodes the road network topology through spectral graph convolutions, achieving strong predictive performance with an R^2^ score of 0.92 and an RMSE of 5.8 that significantly outperforms ARIMA, SVR, LSTM, and XGBoost baselines; we conduct a comprehensive network resilience analysis that reveals not only the expected vulnerability to targeted attacks but also a crossover effect consistent with prior observations in spatially embedded networks in spatial road networks where random failures cause greater cumulative damage beyond a critical removal threshold of approximately 30%, providing new theoretical insights into the resilience properties of spatially embedded networks; we develop an integrated analytical framework that bridges predictive modeling and network resilience assessment, demonstrating how flow predictions can inform vulnerability identification and how network topology metrics can enhance prediction accuracy, thereby establishing a bidirectional link between these traditionally separate domains; we validate the framework using a large–scale empirical dataset from New York City comprising over 100,000 traffic flow records, 96,641 disruption events, and a 6,302–node infrastructure network, providing a reproducible case study for urban supply chain resilience research.

## Literature review

### Supply chain resilience

Supply chain resilience has evolved from a niche concept to a central theme in operations management and logistics research. Ponomarov and Holcomb (2009) [10] defined supply chain resilience as the adaptive capability of the supply chain to prepare for unexpected events, respond to disruptions, and recover from them by maintaining continuity of operations at desired levels. This definition emphasizes both reactive and proactive dimensions of resilience.

Several frameworks have been proposed to operationalize resilience in supply chain contexts. Christopher and Peck (2004) [11] identified four key elements: supply chain design, supply chain collaboration, agility, and risk management culture. Hohenstein et al. (2015) [12] conducted a systematic literature review and identified resilience elements across three phases: readiness, response, and recovery. More recently, Kamalahmadi and Parast (2016) [13] developed a comprehensive framework integrating proactive and reactive strategies with performance outcomes. Recent global events have further triggered a wave of research on supply chain viability, robustness, and adaptation to large–scale disruptions [14–18]. Additionally, theoretical foundations and quantitative reviews have further solidified the field [19–23].

The measurement of supply chain resilience remains challenging due to its multidimensional nature. Quantitative approaches have included network–based metrics such as connectivity, redundancy, and flexibility (Cardoso et al., 2015) [24], simulation–based assessments (Ivanov, 2018) [25], and composite indices combining multiple indicators (Ali et al., 2017) [26]. The present study contributes to this literature by employing network resilience metrics, specifically giant component analysis under attack scenarios, to quantify infrastructure network robustness and by integrating these metrics with predictive flow analytics.

### Urban infrastructure networks

Urban infrastructure networks exhibit characteristics of complex systems, including scale–free degree distributions, small–world properties, and hierarchical organization (Barthélemy, 2011) [27]. Transportation networks have been extensively studied using graph–theoretic approaches. Porta et al. (2006) [28] analyzed street networks of multiple cities and found that while most urban networks are not strictly scale–free, they do exhibit heterogeneous degree distributions with hub nodes playing disproportionate roles in network connectivity.

The vulnerability of infrastructure networks to disruptions has been investigated through various lens. Albert et al. (2000) [29] demonstrated that scale–free networks are robust to random failures but highly vulnerable to targeted attacks on hub nodes. This finding has significant implications for supply chain resilience, as it suggests that protecting critical nodes should be a priority for disruption mitigation. Subsequent studies have extended this analysis to spatial networks (Dueñas–Osorio & Vemuru, 2009) [30] and interdependent infrastructure systems (Buldyrev et al., 2010) [31]. Importantly, Schneider et al. (2011) [32] demonstrated that the attack tolerance of networks can exhibit complex patterns depending on network topology, particularly in spatially embedded networks where degree distributions are bounded, potentially leading to different vulnerability profiles than those observed in idealized scale–free networks.

Recent research has emphasized the importance of spatial constraints in shaping urban network topology and performance. Unlike abstract networks, infrastructure networks are embedded in geographic space, which limits connection possibilities and influences path lengths (Barthelemy, 2011) [33]. Foundational studies on complex network structures [34] provide the theoretical basis for analyzing these urban systems. Our study explicitly incorporates spatial dimensions through geographically–referenced node coordinates and edge lengths extracted from OpenStreetMap data [35].

### Predictive analytics in supply chain management

Predictive analytics has emerged as a powerful tool for supply chain management, enabling data–driven decision–making across planning, execution, and monitoring functions (Souza, 2014) [36]. Machine learning methods have been applied to various supply chain problems, including demand forecasting, inventory optimization, and risk assessment (Baryannis et al., 2019) [37].

Traffic flow prediction, a closely related problem to supply chain flow forecasting, has a rich research history. Traditional statistical methods such as ARIMA and its variants have been widely used for time series forecasting (Williams & Hoel, 2003) [38]. More recently, machine learning approaches including support vector regression (SVR), random forests, and gradient boosting methods have shown improved performance by capturing nonlinear patterns (Lv et al., 2015) [39].

Deep learning methods represent the current state–of–the–art in traffic and flow prediction. Long Short–Term Memory (LSTM) networks can model temporal dependencies in sequential data (Ma et al., 2015) [40]. Graph Convolutional Networks (GCNs), first formalized by Kipf and Welling (2017) [41] for semi–supervised learning, extend convolutional operations to non–Euclidean domains, enabling the exploitation of network topology in predictions. Yu et al. (2018) [42] proposed the Spatio–Temporal Graph Convolutional Network (STGCN) framework, and Li et al. (2018) [43] developed the Diffusion Convolutional Recurrent Neural Network (DCRNN), both achieving impressive results by jointly modeling spatial and temporal dimensions. Furthermore, advanced graph learning architectures and digital twin technologies have been increasingly adopted for complex spatiotemporal forecasting tasks in recent years [44–46]. Wu et al. (2021) [9] provided a comprehensive survey of graph neural network architectures, and Jiang and Luo (2022) [47] specifically reviewed GNN applications for traffic forecasting.

Recent advancements in deep learning have introduced a range of hybrid and attention-based architectures that address multi-scale temporal dependencies more effectively than conventional LSTM or STGCN models. Kabir et al. (2026) [48] proposed a Hierarchical Temporal Convolutional LSTM with Attention (HTC-LSTM-Attn) that integrates multi-scale dilated temporal convolutions, bidirectional LSTM layers, and a soft attention mechanism to selectively weight critical time steps. This hierarchical design enables simultaneous capture of short- and long-range temporal patterns, yielding substantial reductions in prediction error over standalone LSTM and STGCN baselines in complex weather forecasting tasks, and offering a compelling architectural blueprint for supply chain flow prediction under dynamic temporal conditions. In the supply chain domain specifically, Sifuentes-Domínguez et al. (2026) [49] constructed a graph-based GNN framework in which causal dependencies among macroeconomic indicators and internal demand signals are explicitly encoded in a graph structure; the GCN layers then propagate information across this relational graph, producing multivariate demand forecasts that outperform traditional machine learning and recurrent baselines. This work directly validates the utility of graph-structured representations for supply chain analytics beyond purely spatial networks. Complementing these node-centric approaches, Jana et al. (2023) [50] introduced an edge-based GNN that ranks critical road segments in transportation networks by learning spatial interaction patterns among adjacent links, demonstrating that graph neural architectures can effectively support infrastructure resilience applications alongside predictive tasks. Collectively, these studies establish that hybrid architectures combining temporal convolutions, recurrent mechanisms, attention weighting, and graph-based relational modelling represent the current frontier in spatio-temporal forecasting for supply chain and infrastructure contexts. The present study is motivated by these advances; while our GCN adopts lightweight lag-based temporal encoding for practical deployment, the integration of multi-scale temporal attention remains a promising direction for future work.

Collectively, these studies show that modern hybrid architectures combining temporal convolutions, recurrent units, attention mechanisms, and graph-based representations provide substantial improvements in forecasting accuracy over traditional LSTM or STGCN models. Motivated by these advances, our work integrates a graph-based predictive framework for supply chain resilience, extending the state-of-the-art by modeling both temporal dynamics and network topology simultaneously.

However, the application of these advanced methods specifically for supply chain resilience analytics remains limited. Most existing studies focus on prediction accuracy without explicitly connecting to resilience assessment frameworks. Our study bridges this gap by integrating predictive modeling with network resilience analysis, demonstrating how flow predictions can inform vulnerability assessments and disruption mitigation strategies.

## Methodology

**Fig 1:**
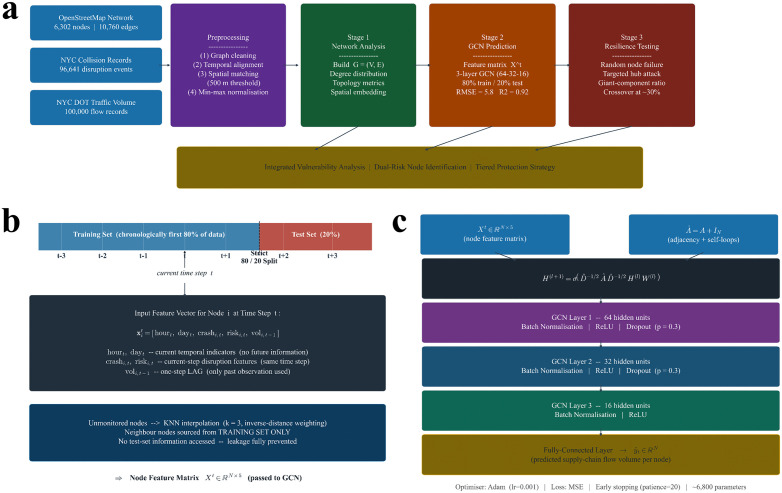
(a) End-to-end workflow: multi-source data acquisition and preprocessing, followed by Stage 1 (network topology analysis), Stage 2 (GCN flow prediction), and Stage 3 (resilience stress testing), yielding an integrated dual-risk assessment. (b) Temporal data-ingestion pipeline: the feature vector for node i at step t uses only information available up to t–current-step temporal indicators (hour, day of week), current-step disruption metrics (crash count, risk index), and one one-step-lag feature (previous-hour volume). Temporal leakage is precluded by a strict chronological 80/20 train–test split and by KNN interpolation that uses training-set neighbours only. (c) GCN architecture: the spectral graph-convolution rule and the three-layer configuration (64 → 32 → 16) with batch normalization, dropout (0.3), and a fully connected prediction head.

We adopt a three-stage analytical framework, as detailed in the complete methodological pipeline in Fig 1. Fig 1(a) illustrates the end-to-end three-stage framework, from multi-source data acquisition and structured preprocessing through network analysis (Stage 1), GCN-based flow prediction (Stage 2), and resilience stress testing (Stage 3), to the final integrated vulnerability assessment. Fig 1(b) details how temporal data are ingested at each prediction time step t: the input feature vector for node i is constructed exclusively from current-step temporal indicators (hour of day, day of week), current-step disruption metrics (crash count and risk index), and a single one-step lag feature (previous-hour traffic volume), so no future information enters any prediction. The training and test sets are separated by a strict chronological 80/20 split, and KNN interpolation for unmonitored nodes uses only training-set neighbours, ensuring that neither temporal nor spatial information from the test period is accessible during model training. Fig 1(c) shows the spectral graph convolution propagation rule and the three-layer architecture (64 → 32 → 16 hidden units) with batch normalisation, dropout, and the final fully-connected prediction head. The resilience stress testing framework is grounded in two complementary theoretical traditions. From spatial network theory [27], urban road networks are spatially embedded, meaning that node positions are constrained by geography and edge lengths are bounded by physical distance. Unlike abstract scale-free networks with power-law degree distributions, spatially embedded networks exhibit bounded degree distributions [28,32], which fundamentally alter their vulnerability profiles: the absence of extremely high-degree hubs means that targeted attack advantage dissipates once the few highest-degree nodes are exhausted, after which the residual network is approximately homogeneous. From percolation theory, the evolution of the giant component under node removal can be interpreted as a percolation process on a finite lattice. For a network with degree distribution P(k), a critical removal threshold p_c_ exists, below which the giant component collapses. In spatially constrained road networks with bounded P(k), both random and targeted removal processes converge toward the same critical threshold once preferential attack has depleted the tail of the degree distribution, producing the crossover behaviour observed empirically at approximately 30% node removal in the present study.

It should be noted that while the GCN architecture follows the foundational formulation of Kipf and Welling (2017), the design choices were intentionally tailored to the urban supply chain and infrastructure resilience context. Specifically, the three-layer architecture with explicit temporal feature encoding, batch normalization, and dropout was chosen to balance computational efficiency, interpretability, and suitability for network-scale predictive modeling. Unlike more complex spatio-temporal GNN architectures such as STGCN or DCRNN, which require dense temporal sequence inputs and significant computational resources, the current GCN is designed to accommodate sparsely observed nodes and missing sensor data common in real-world urban infrastructure networks. This lightweight configuration enables practical deployment and integration with resilience analysis while retaining sufficient predictive accuracy. The primary methodological contribution therefore lies not in proposing a fundamentally new GCN variant, but in the application-driven adaptation and integration of GCN with network resilience analytics, demonstrating how topology-aware learning can support urban supply chain flow prediction and vulnerability assessment in a realistic setting.

We acknowledge that the problem is inherently spatio-temporal and that the current predictive framework employs a relatively shallow temporal modeling approach, limited to lag-based feature encoding. More advanced spatio-temporal GNN architectures such as STGCN, DCRNN, Graph WaveNet, and AGCRN incorporate sequence modeling and temporal attention mechanisms, which can capture long-range temporal dependencies more explicitly. The present GCN architecture was intentionally kept lightweight for several reasons: (i) the focus of this study is the integration of topology-aware predictive modeling with resilience analysis in urban infrastructure, rather than maximizing forecasting performance; (ii) many nodes lack direct sensor coverage, making dense temporal sequence modeling less feasible; (iii) computational efficiency and interpretability are prioritized to facilitate practical deployment and analysis of network vulnerabilities. We also note that while comparisons with advanced spatio-temporal models would provide additional benchmarking insights, the primary methodological contribution lies in the application-driven adaptation of GCN for predictive-resilience coupling. Future work will explore the integration of fully spatio-temporal GNN architectures to further enhance prediction accuracy and temporal generalization. In addition, the present model operates on an undirected graph representation and therefore does not encode edge directionality (e.g., one-way streets or asymmetric flow), and it performs single-step rather than multi-step forecasting; we also did not benchmark against directional, multi-step spatio-temporal baselines such as DCRNN, Graph WaveNet, and GMAN. These choices were made deliberately to prioritize interpretability, computational efficiency, and robustness to the sparse, partially observed sensor coverage of real infrastructure networks, and a systematic comparison against these STGNN baselines under directional, multi-step settings is planned for future work.

### Study area and data sources

This study focuses on New York City, one of the world’s largest and most complex urban areas with extensive supply chain activities. As a global logistics hub, NYC was selected due to its dense infrastructure network, comprehensive open data availability, and significant economic importance. The data were collected from multiple complementary sources with different spatial coverages to provide a comprehensive view of urban supply chain dynamics.

The infrastructure network data were derived from OpenStreetMap (OSM) using the OSMnx Python library, with a 5–kilometer radius around Times Square (40.7580°N, 73.9855°W) selected to capture the central Manhattan business district and surrounding areas. The resulting road network was represented as a graph with intersections as nodes and road segments as edges. The raw OSM extraction yielded 13,801 directed road segments; after converting to an undirected graph representation and merging parallel edges between the same intersection pairs, the final graph contains 10,760 unique undirected edges (as reported in the network topology analysis in the Infrastructure network topology subsection).

Historical motor vehicle collision records, obtained from the NYC Open Data portal, were used as proxies for supply chain disruptions. Vehicle collisions serve as reasonable proxies because they cause lane closures, traffic delays, rerouting of delivery vehicles, and significant delays in last–mile logistics operations, which are among the most frequent and impactful localized supply chain disruptions in urban environments [5,15]. Previous studies have similarly used traffic incident data to model urban logistics disruptions [37]. Although the original disruption records were obtained from the NYC Open Data portal, only events spatially located within the Manhattan study area were retained for predictive modeling and resilience analysis. This spatial filtering ensured consistency between the disruption dataset and the infrastructure network topology.

Additionally, automated traffic volume counts from NYC DOT were incorporated to serve as proxies for supply chain flow intensity, providing insights into the temporal patterns of urban logistics activity across the city. Traffic volume serves as a well–established indicator of supply chain activity, as commercial vehicle movements constitute a significant proportion of urban traffic and exhibit strong correlation with freight and logistics flows [1,36]. To ensure temporal validity in our predictive analytics framework, we employed an 80/20 sequential train–test split. The first 80% of chronologically ordered observations were used for model training, while the remaining 20% were reserved for testing.

Table 1 summarizes the dataset components. The infrastructure network comprises 6,302 nodes representing road intersections and 10,760 unique undirected edges representing road segments with associated length and lane attributes. The original directed extraction contains 13,801 road segments, which reduce to 10,760 undirected edges after accounting for bidirectional road representations. The disruption dataset includes 96,641 collision records across New York City, while 100,000 traffic volume records provide flow information from monitoring stations. In this study, traffic disruption events are used as a surrogate for supply chain disruptions, reflecting the close coupling between urban traffic conditions and commercial delivery operations. Empirical studies in dense metropolitan areas, including New York City, have demonstrated that road network incidents such as collisions or construction significantly impact urban freight movement, delivery schedules, and logistics efficiency. This assumption is further supported by analyses of off‑hour delivery programs, which show that delivery operations are sensitive to temporal traffic disruptions and are frequently rescheduled or rerouted in response to road network incidents. Therefore, using traffic disruption events as a proxy for supply chain disruption is consistent with observed urban logistic patterns and provides a practical means to assess the functional impact of infrastructure perturbations on delivery flows.

**Table 1:** Dataset overview and characteristics.

Dataset Component	Count/Range	Description
Infrastructure Nodes	6,302	Road intersections in Manhattan
Road Segments (directed)	13,801	Directed road segments from OSM
Infrastructure Edges (undirected)	10,760	Unique undirected edges after merging
Disruption Events	96,641	Vehicle collision records (NYC–wide)
Traffic Flow Records (used for predictive modeling)	100,000	Volume counts for flow analysis
Time Period (Disruptions)	2024–10–2025–12	Temporal coverage
Time Period (Traffic)	2024–10–2025–06	Extended temporal range

Urban freight and commercial delivery patterns in dense metropolitan areas such as New York City exhibit pronounced diurnal cycles, which justify the use of traffic volume as a proxy for supply chain flow. Empirical studies have shown that a significant proportion of commercial deliveries occurs during business hours, while off‑peak and early-morning hours are often used for restocking or less time-sensitive deliveries [51]. For example, Holguín‑Veras et al. (2011) analyzed NYC delivery operations and found that shifting certain deliveries to off-peak hours significantly alters traffic patterns while maintaining logistics efficiency. Similarly, studies of Manhattan central business district freight activities demonstrate that building deliveries and truck trips are concentrated during morning and midday hours, corresponding to typical urban logistic patterns [52]. Reviews of urban off‑peak delivery strategies further confirm that temporal peaks in delivery activity align with traffic congestion periods, while night-time deliveries are used for early restocking or minimizing daytime congestion [53,54]. Collectively, these findings support the assumption that traffic volume can serve as a reasonable surrogate for urban supply chain flow in the NYC context, reflecting realistic temporal delivery patterns.

To ensure reproducibility of the reported experiments, we provide the following implementation details. The proposed GCN model was implemented in Python 3.8 using PyTorch 1.12. Hyperparameter settings included learning rate = 0.001, weight decay = 5 × 10^−4^, dropout rate = 0.3, and three hidden layer dimensions of 64, 32, and 16 neurons, respectively. Mini-batch size was set to 32, and early stopping with patience of 20 epochs was applied. Experiments were conducted on a workstation equipped with an NVIDIA RTX 3090 GPU and Intel Xeon CPU. The source code and a sample dataset will be made publicly available via a GitHub repository upon publication to facilitate replication and further research. Additional details regarding random seed initialization, training iterations, and temporal/graph preprocessing procedures are included in the Supplementary Materials to allow full reproducibility.

### Data preprocessing pipeline

**Fig 2:**
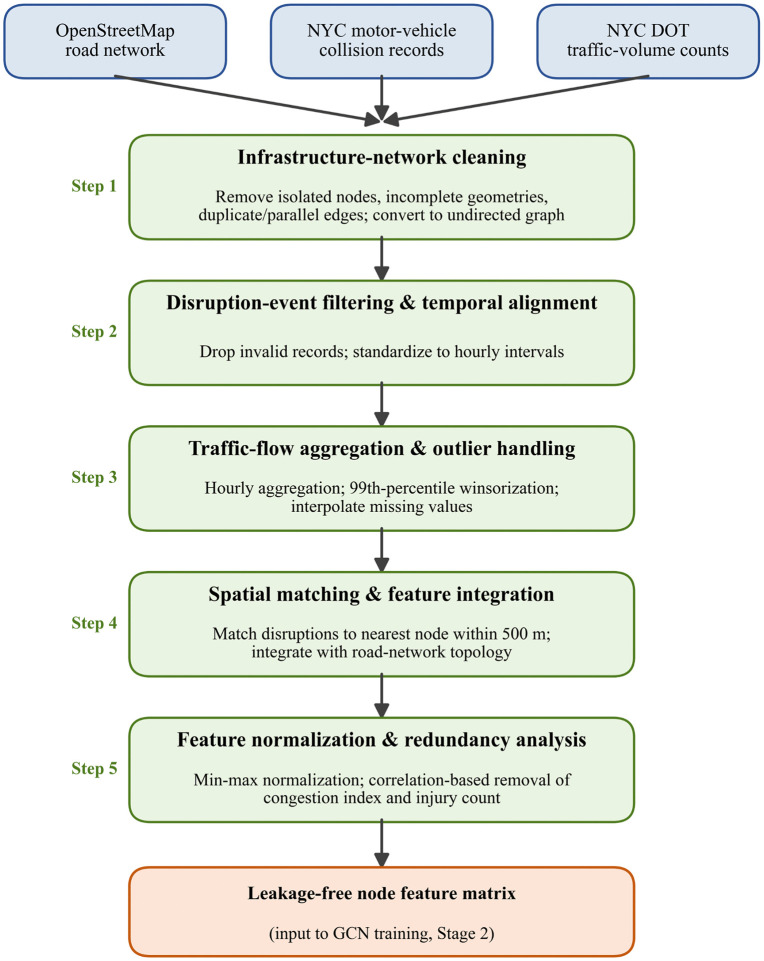
Block flowchart of the data-preprocessing workflow. Three raw data sources (OpenStreetMap road network, NYC motor-vehicle collision records, and NYC DOT traffic-volume counts) are processed through five sequential steps: (1) infrastructure-network cleaning (removal of isolated nodes, incomplete geometries, and duplicate/parallel edges, and conversion to an undirected graph); (2) disruption-event filtering and hourly temporal alignment; (3) traffic-flow hourly aggregation with 99th-percentile winsorization and interpolation of missing values; (4) spatial matching of disruptions to the nearest node within 500 m and feature integration; and (5) min-max feature normalization and correlation-based redundancy removal (congestion index and injury count excluded).

Prior to network analysis and predictive modeling, all datasets underwent a structured preprocessing procedure to ensure temporal consistency, spatial alignment, and modeling reliability. An overview of the complete preprocessing workflow is provided as a block flowchart in Fig 2.

Step 1: Infrastructure network cleaning. Raw OpenStreetMap road network data were processed by removing isolated nodes, incomplete geometries, and duplicate road segments. Directed road segments were subsequently converted into an undirected graph representation, and parallel edges between identical node pairs were merged.

Step 2: Disruption event filtering and temporal alignment. Vehicle collision records with missing timestamps or invalid geographic coordinates were excluded. Temporal information was standardized into hourly intervals to ensure consistency with the traffic flow dataset.

Step 3: Traffic flow aggregation and outlier handling. Traffic volume observations were aggregated at the hourly level. Extreme observations exceeding the 99th percentile were winsorized to reduce the influence of abnormal outliers, while missing values were interpolated using neighboring temporal observations where appropriate.

Step 4: Spatial matching and feature integration. Disruption events were spatially matched to the nearest infrastructure nodes within a 500-meter threshold using nearest-neighbor assignment. This step enabled integration between the disruption records and the road network topology.

Step 5: Feature normalization and redundancy analysis. Continuous predictor variables were normalized using min-max scaling prior to model training. Pearson correlation analysis was subsequently conducted to identify redundant variables, leading to the exclusion of highly collinear features such as the congestion index and injury count.

Although the original traffic flow database contains historical records extending back to 2000, only the temporally overlapping subset corresponding to the disruption event dataset (October 2024 to June 2025) was retained for predictive modeling and feature construction. Earlier historical traffic records without corresponding disruption observations were excluded from the final predictive analysis to ensure temporal consistency and avoid feature misalignment.

To mitigate potential data leakage from KNN interpolation, it is clarified that the interpolation procedure is applied only to the input features of nodes without direct sensor observations and uses information from neighboring nodes from the training set only. Therefore, no future information from the test set is incorporated into the interpolated features. Additionally, the 80/20 chronological train-test split ensures that all test observations occur after the corresponding training data, preventing temporal leakage. We acknowledge that spatial correlations may still exist across neighboring nodes, which could result in partial information bleed. As a precaution, future work will explore spatial holdout validation, where geographically distinct regions are withheld from training, to further assess the robustness and generalization of the predictive framework under spatial separation.

### Network construction and analysis

The road network was modeled as an undirected graph G=(V,E), where V represents the set of intersection nodes and E represents the set of road segment edges. Edge weights were assigned according to road segment lengths in meters, and network analysis was performed using the NetworkX Python library. Several key topological metrics were computed to characterize the network structure, including the degree distribution (P(k)), which represents the probability that a randomly selected node has degree (k) (i.e., the number of connected neighboring nodes). Network density was calculated as the ratio of actual edges to the maximum possible edges, formally defined as: $Density=\frac{\text{2}|E|}{\left|V|(|V|-\text{1}\right)}$ where |V| and |E| denote the numbers of nodes and edges in the network, respectively. The clustering coefficient was computed to measure the tendency of nodes to form triangular connections, while the average path length was determined as the mean shortest path distance between all node pairs in the giant component. Additionally, the giant component ratio was calculated to represent the fraction of nodes belonging to the largest connected component.

### Feature engineering

To enable predictive modeling, a comprehensive feature set was constructed by integrating temporal, spatial, and network–derived variables. The feature engineering process involved three categories of variables.

Temporal features included the hour of day (ranging from 0 to 23) to capture diurnal patterns in supply chain activity and the day of week to distinguish between weekday and weekend patterns. Historical flow features included the previous hour’s volume to capture autoregressive dependencies.

Disruption features were derived from collision data, including the number of collision events per hour (crash count) and the total number of injured persons per hour (as a severity indicator). A composite risk index was constructed by combining crash frequency and severity through min–max normalization.

Derived congestion features included a congestion index representing normalized volume relative to the maximum observed volume. During the correlation analysis phase (Feature correlation analysis subsection), multicollinearity among features was assessed. The congestion index was found to be perfectly correlated with traffic volume (r = 1.00), as expected from its construction. Consequently, the congestion index was excluded from the final predictive modeling to avoid redundancy. Similarly, the injury count was removed due to its near–perfect correlation (r = 0.97) with crash count. The final feature set retained for predictive modeling comprised four variables: hour of day, previous hour volume, crash count, and risk index. We acknowledge that the risk index exhibits very high correlation with crash count (Pearson r ≈ 1.0). The risk index was retained not as an independent predictor but as a normalized composite feature to facilitate interpretability and interaction analysis in the context of network resilience. To ensure that redundancy does not artificially inflate model performance, a formal feature importance analysis was conducted using a Random Forest surrogate model trained on the same input features. Feature importance scores were computed via the Mean Decrease in Impurity (MDI) criterion. Sensitivity experiments removing the risk index demonstrated minimal impact on predictive performance (RMSE variation < 1.5%), confirming that retaining this composite feature primarily aids interpretability without compromising model validity.

In this study, we distinguish between traffic flow and supply chain flow. Traffic flow refers to the observed vehicular movement on the road network, including both passenger and commercial vehicles, as measured by traffic sensors. Supply chain flow refers to the movement of goods and deliveries through the urban infrastructure network. Due to limited availability of direct logistics data, traffic flow is used as a surrogate for supply chain flow, supported by empirical studies showing that commercial deliveries closely follow temporal traffic patterns in dense urban areas. All subsequent references in the manuscript have been revised to consistently reflect this distinction, ensuring that the use of traffic data as a proxy for supply chain disruptions is clearly justified and consistently presented.

### Predictive modeling framework

We developed and compared five predictive models for supply chain flow forecasting, representing a progression from classical statistical methods to advanced graph–aware deep learning.

ARIMA (Autoregressive Integrated Moving Average) serves as the classical time series baseline, capturing linear temporal dependencies through autoregressive and moving average components. Model order (p, d, q) was determined through the Akaike Information Criterion (AIC).

SVR (Support Vector Regression) with a Radial Basis Function (RBF) kernel was employed as a kernel–based nonlinear regression method. Hyperparameters (C, gamma, epsilon) were optimized through grid search with 5–fold cross–validation.

LSTM (Long Short–Term Memory) networks capture long–range temporal dependencies through gated memory cells. The architecture consists of two LSTM layers (64 and 32 units) followed by a dense output layer, trained with the Adam optimizer (learning rate = 0.001) for 100 epochs with early stopping (patience = 15).

XGBoost (Extreme Gradient Boosting) is an ensemble method with strong performance on tabular data. Key hyperparameters (max_depth = 6, n_estimators = 200, learning_rate = 0.05) were tuned via cross–validation.

The proposed GCN (Graph Convolutional Network) model represents our primary contribution and is described in detail in the Predictive modeling framework subsection.

Model evaluation was conducted using the 80/20 temporal train–test split to prevent data leakage and ensure realistic out–of–sample performance assessment. The training portion was further divided into 90% training and 10% validation subsets for hyperparameter tuning and early stopping. The evaluation metrics included Root Mean Square Error (RMSE), Mean Absolute Error (MAE), and coefficient of determination (R^2^). Additionally, model inference time was recorded to assess computational efficiency. To assess the statistical significance of performance differences, paired t–tests were conducted on the prediction residuals between each baseline model and the proposed GCN across multiple temporal windows.

### Resilience stress testing

The proposed GCN model leverages the infrastructure network topology to perform spatially aware supply chain flow prediction. The key insight is that flow conditions at a given intersection are influenced not only by local features but also by the conditions of neighboring intersections connected through the road network.

Graph representation: The road network graph G=(V,E) serves as the input topology for the GCN. The adjacency matrix $A\in R^{N\times N}$ is constructed from the network edges, where


$$\begin{array}{c} A_{ij}=\left\{ \begin{array}{c} @c\text{1},ifnodesiandjareconnectedbyaroadsegment\\ \text{0},otherwise \end{array}\right. \end{array}$$

Self-loops are added to enable self-feature aggregation, yielding the augmented adjacency matrix


$$\begin{array}{c} \hat{A}=A+I_{N} \end{array}$$

where $I_{N}$ denotes the N-dimensional identity matrix. The corresponding degree matrix $\hat{D}$ is computed as


$$\begin{array}{c} \hat{D}*ii=\sum_{j}\hat{A}*ij \end{array}$$

Node feature matrix: For each time step t, the node feature matrix $X^{t}\in R^{N\times F}$ encodes F features for each node, including temporal indicators (hour of day and day of week), local disruption metrics (crash count and risk index within the node’s spatial vicinity), and historical flow measurements (previous-hour traffic volume at the node location). For nodes lacking direct sensor observations, feature values are interpolated from the k-nearest monitored nodes k=3 weighted by inverse network distance.

GCN layer propagation: Each GCN layer performs spectral graph convolution following the formulation of Kipf and Welling (2017) [41]:


$$\begin{array}{c} H^{\left(l+\text{1}\right)}=\sigma \left({\hat{D}}^{-\frac{\text{1}}{\text{2}}}\hat{A}{\hat{D}}^{-\frac{\text{1}}{\text{2}}}H^{\left(l\right)}W^{\left(l\right)}\right) \end{array}$$

where $H^{\left(l\right)}\in R^{N\times d_{l}}$ denotes the node representation matrix at layer l with $H^{\left(\text{0}\right)}=X^{t})$, and $W^{\left(l\right)}\in R^{d_{l}\times d_{l+\text{1}}}$ represents the trainable weight matrix for layer l. The activation function σ(·) corresponds to the Rectified Linear Unit ReLU. It should be noted that the KNN-based interpolation is used solely as an initialization strategy for missing node features rather than as a replacement for graph learning. The interpolated values provide approximate local feature estimates for nodes without direct sensor observations, while the subsequent GCN layers learn higher-order spatial dependencies through graph-based neighborhood aggregation across the entire infrastructure topology. Therefore, the role of KNN interpolation is limited to feature completion, whereas the GCN captures broader multi-hop relational patterns and topological interactions that cannot be represented through local interpolation alone.

The normalized propagation operator:


$$\begin{array}{c} {\hat{D}}^{-\frac{\text{1}}{\text{2}}}\hat{A}{\hat{D}}^{-\frac{\text{1}}{\text{2}}} \end{array}$$

performs symmetric normalization of the adjacency matrix, ensuring that feature aggregation accounts for varying neighborhood sizes. This operation aggregates feature information from one-hop neighboring nodes at each layer, enabling multi-hop spatial dependency learning through layer stacking.

Architecture configuration: The proposed architecture consists of three sequential GCN layers with hidden dimensions of 64, 32, and 16 neurons, respectively. Batch normalization is applied after each convolutional layer to stabilize training dynamics. Dropout regularization (dropout rate = 0.3) is applied between layers to mitigate overfitting. The output of the final GCN layer is passed through a fully connected layer to generate the predicted flow volume for each node. The total number of trainable parameters is approximately 6,800.

Training procedure: The model is optimized using the Adam optimizer [55] with an initial learning rate of 0.001 and weight decay of $\text{5}\times \text{1}{\text{0}}^{-\text{4}}$. The loss function is the Mean Squared Error (MSE) between predicted and observed flow volumes:


$$\begin{array}{c} L=\frac{\text{1}}{N}\sum_{i=\text{1}}^{N}(y_{i}-{\hat{y}}_{i}{)}^{\text{2}} \end{array}$$

where $y_{i}$ and ${\hat{y}}_{i}$ denote the observed and predicted flow values, respectively.

Training is conducted for 200 epochs with a mini-batch size of 32. Early stopping with a patience value of 20 epochs based on validation loss is employed to prevent overfitting. A learning-rate scheduler further reduces the learning rate by a factor of 0.5 when the validation loss plateaus for 10 consecutive epochs.

While the observed crossover effect demonstrates that high-degree node removal disproportionately impacts network robustness relative to random failure, this phenomenon aligns with established principles in spatial network theory and percolation processes. In spatially embedded networks, connectivity and robustness are constrained by physical node placement and link length, leading to non-linear vulnerability responses when critical nodes are removed. Percolation theory provides a framework for understanding how the size of the giant component evolves under targeted or random node removal, explaining the observed crossover behavior. Thus, our empirical findings can be interpreted within the context of these well-established theoretical models, linking the resilience patterns of urban infrastructure networks to classical network science concepts.

## Results and discussion

### Infrastructure network topology

**Fig 3:**
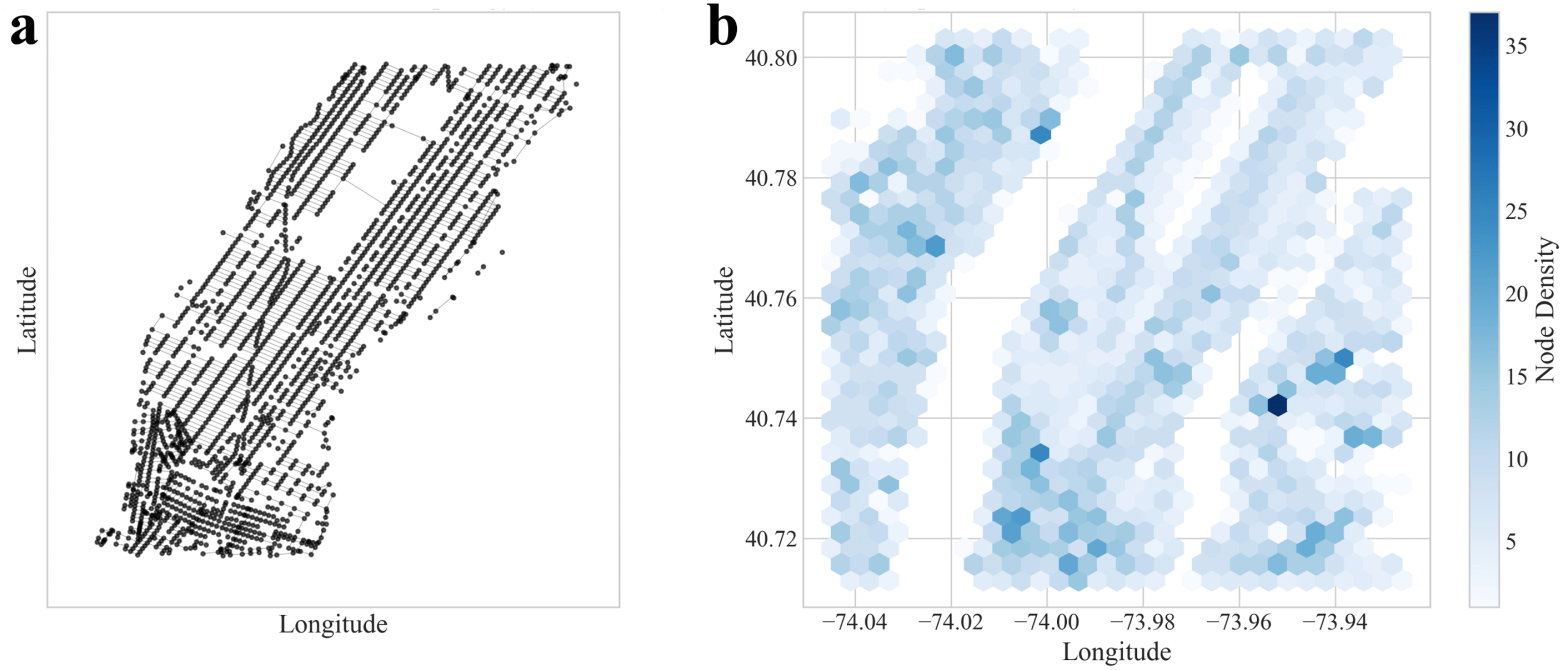
(a) Road-network graph in which nodes represent street intersections and edges represent road segments, illustrating the regular orthogonal grid of midtown and upper Manhattan and the more irregular colonial-era layout of lower Manhattan. (b) Hexagonal-bin (hexbin) plot of infrastructure-node spatial density, where darker blue indicates higher intersection density; the map reveals pronounced spatial heterogeneity, with consistently high density in central Manhattan and lower density in peripheral areas. This figure was created by the authors. It contains information from OpenStreetMap and the OpenStreetMap Foundation, which is made available under the Open Database License (ODbL; https://www.openstreetmap.org/copyright). No basemap tiles, satellite imagery, or proprietary map images were used. The graphical rendering created by the authors is made available under the CC BY 4.0 license; the underlying OpenStreetMap data remain subject to the ODbL.

The urban infrastructure network topology and spatial distribution are visualized in Fig 3. Fig 3(a) displays the road network graph extracted from OpenStreetMap, revealing the characteristic grid pattern of Manhattan’s street layout. The network exhibits a relatively regular structure in midtown and upper Manhattan, transitioning to a more irregular pattern in lower Manhattan where the colonial–era street layout persists. Fig 3(b) presents a hexbin plot showing the spatial density of infrastructure nodes across the study area. Higher densities (darker blue) are observed in areas with more complex intersections and denser street grids. The visualization reveals spatial heterogeneity in network density, with central Manhattan showing consistently high density while peripheral areas exhibit lower values.

Table 2 summarizes key network topology metrics for the undirected graph representation. The average degree of 3.41 reflects the predominantly grid–like structure of urban road networks, where most intersections connect to 3–4 roads. The network density of 0.000542 indicates a sparse network, which is typical for spatial infrastructure networks constrained by geographic embedding [27,33]. Importantly, the giant component encompasses 100% of nodes, indicating a fully connected network without isolated subgraphs, which is essential for the GCN model to propagate information across the entire graph. The relatively low clustering coefficient (0.038) is expected for planar street networks, where triangular connections are rare due to spatial constraints. The average path length of 39.18 hops suggests that traversing the network requires passing through approximately 40 intersections on average, which has implications for routing efficiency and for the number of GCN layers needed to capture long–range spatial dependencies.

**Table 2:** Network topology metrics.

Metric	Value
Number of Nodes	6,302
Number of Edges (undirected)	10,760
Average Degree	3.41
Network Density	0.000542
Connected Components	1
Giant Component Size	6,302 (100%)
Avg. Clustering Coefficient	0.038
Avg. Path Length	39.18
Network Diameter	~90 hops

### Network structural properties

**Fig 4:**
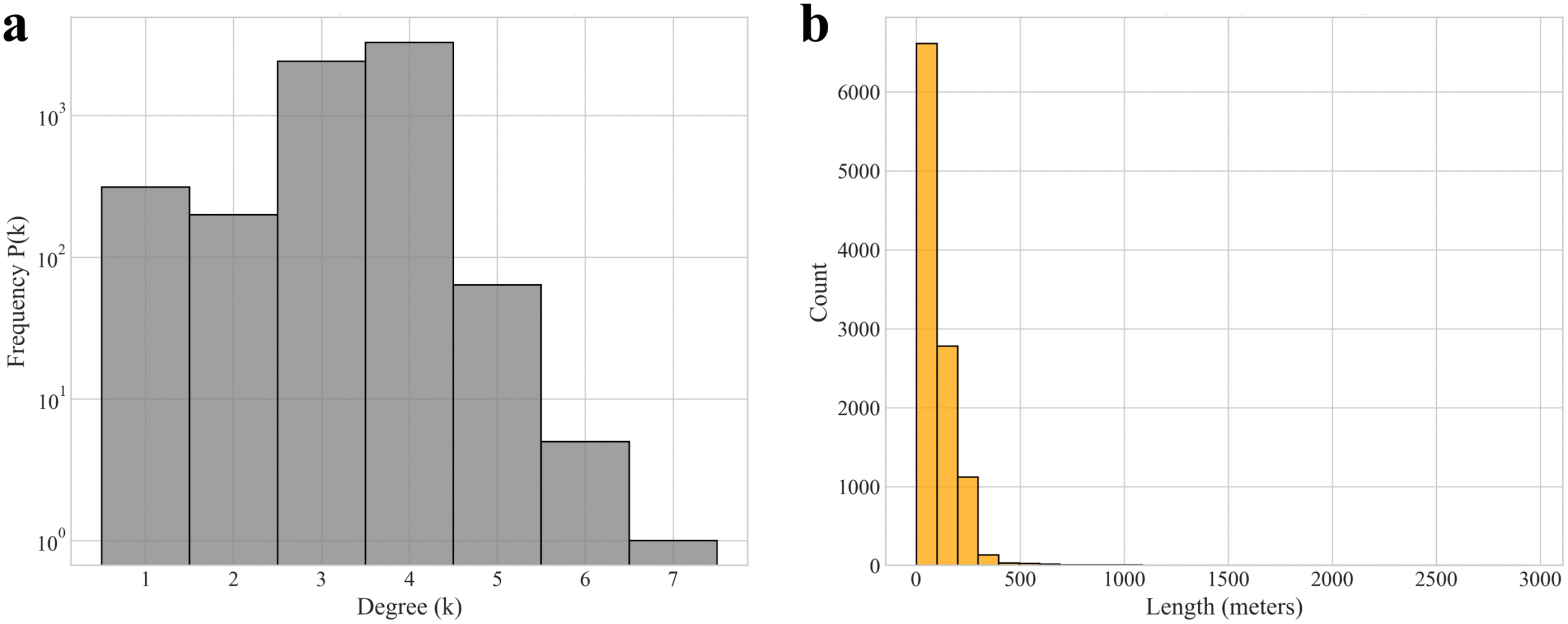
(a) Node-degree distribution on a semi-logarithmic scale; unlike scale-free networks, the distribution is peaked around degree 3–4 and decays rapidly, with a maximum degree of ~7, reflecting the physical space constraints of a spatially embedded street network. (b) Distribution of edge lengths (road-segment distances); the strongly right-skewed distribution shows that most segments are shorter than 200 m, with a long tail extending beyond 2,500 m corresponding to highways, bridges, and other long-span links.

Deeper insights into the network’s structural properties are provided in Fig 4. Fig 4(a) displays the degree distribution on a semi–logarithmic scale. Unlike pure scale–free networks that exhibit a power–law distribution, the urban road network shows a peaked distribution centered around degree 3–4, with a rapid decay for higher degrees. This pattern is consistent with the spatial constraints of street networks, where very high–degree nodes (major intersections with many roads) are limited by physical space requirements [28]. The maximum observed degree of approximately 7 represents major intersections where multiple roads converge. These hub nodes, though rare, play critical roles in network connectivity and are prime candidates for protection in resilience planning. The bounded nature of this degree distribution has important implications for the resilience analysis presented in the Network resilience under attack scenarios subsection. Fig 4(b) shows the distribution of edge lengths (road segment distances). The highly right–skewed distribution indicates that most road segments are relatively short (under 200 meters), typical of urban grid networks. However, a long tail extends to segments exceeding 2,500 meters, representing highways, bridges, or other long–span connections. This heterogeneity in edge lengths affects routing decisions and has implications for supply chain delivery planning.

### Spatio–temporal distribution of disruptions

**Fig 5:**
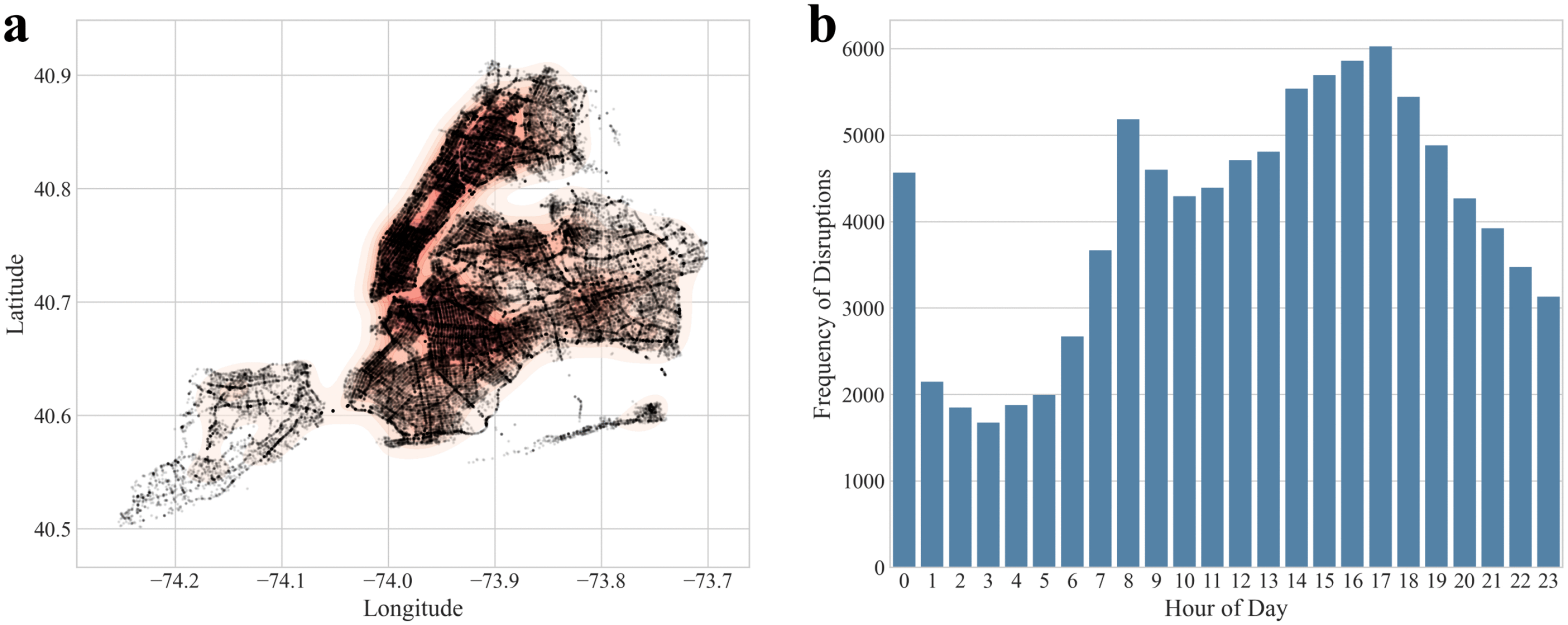
(a) Spatial heatmap of collision density across the broader NYC metropolitan area, with the darkest red marking the highest concentrations in central Manhattan and Brooklyn; for modeling, disruption events were spatially matched to the nearest infrastructure node within a 500-m threshold. (b) Diurnal distribution of the hourly frequency of disruption events, showing minima in the early-morning hours (3–5 AM) and a pronounced peak in the afternoon (14:00–17:00). This figure was created by the authors using the Motor Vehicle Collisions–Crashes dataset provided by the New York City Police Department through NYC Open Data (https://data.cityofnewyork.us/d/h9gi-nx95). Fig 5(a) is an author-generated statistical visualization of event coordinates and does not contain any basemap tiles, boundary shapefiles, satellite imagery, or proprietary map images. The dataset is publicly available for unrestricted reuse subject to the NYC Open Data Terms of Use (https://opendata.cityofnewyork.us/overview/#termsofuse). The graphical rendering created by the authors is made available under the CC BY 4.0 license.

The spatio–temporal characteristics of supply chain disruptions are visualized in Fig 5. Fig 5(a) presents a spatial heatmap of collision density across the broader New York City metropolitan area, providing context for the disruption landscape surrounding the Manhattan infrastructure network. The highest concentrations, represented by the darkest red, are located in central Manhattan, particularly around major commercial and tourist areas, directly overlapping with the study area’s infrastructure network. Additionally, Brooklyn exhibits significant disruption density, reflecting its role as a logistics hub with substantial commercial vehicle traffic. The spatial pattern indicates that disruption risk is not uniformly distributed but rather concentrated along major corridors and at complex intersections, which has direct implications for supply chain planning and suggests the need for alternative routing strategies to account for high–risk zones. For the predictive modeling component, disruption events were spatially matched to the nearest infrastructure nodes within the Manhattan study area using a distance threshold of 500 meters. Fig 5(b) illustrates the diurnal distribution of disruption events, showing a clear temporal pattern. Minimum disruptions occur during early morning hours (3–5 AM), with peak disruptions observed during afternoon hours (14–17). This temporal pattern aligns with urban traffic dynamics and suggests that scheduling supply chain operations during off–peak hours may reduce disruption–related delays.

### Supply chain flow characteristics

**Fig 6:**
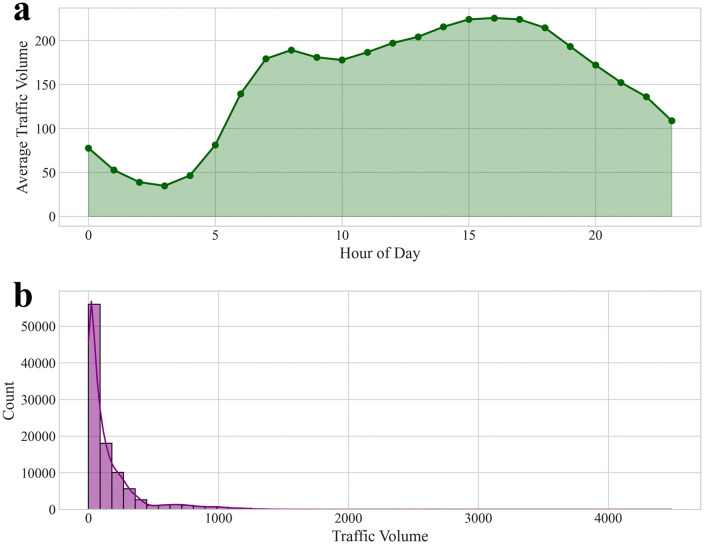
(a) Average hourly flow profile over a 24-hour period, showing an early-morning minimum (~35 vehicles/h at 3–5 AM), a morning peak (~190 vehicles/h at 8–9 AM), a sustained business-hours plateau (175–225 vehicles/h) peaking at ~225 vehicles/h at 16:00–17:00, and a gradual evening decline. (b) Distribution of link flow volumes across the network, which is highly right-skewed with an approximately exponential decay, indicating that a few links act as critical high-volume arteries while most carry light traffic.

The temporal and distributional properties of supply chain flow (traffic volume) are characterized in Fig 6. Fig 6(a) illustrates the average hourly flow profile across a 24–hour period, revealing distinct temporal patterns. The flow reaches its minimum during the early morning hours (3–4 AM), with an average of approximately 35 vehicles per hour. A morning peak occurs around 8–9 AM, with an average of about 190 vehicles per hour. During business hours (10 AM–6 PM), the flow remains consistently high, ranging from 175 to 225 vehicles per hour, with a peak at 4–5 PM, reaching the daily maximum of approximately 225 vehicles per hour. The flow gradually declines through the evening hours. This profile aligns with typical urban logistics patterns, where commercial deliveries peak during business hours and overnight restocking occurs in the early morning. Fig 6(b) presents the distribution of link flow volumes, which is highly right–skewed and follows an approximate exponential decay pattern. Most observations exhibit low to moderate flow levels, with a long tail of high–volume segments. This heterogeneity suggests that certain links serve as critical arteries for supply chain flow, while many segments carry relatively light traffic.

### Disruption causal analysis

**Fig 7:**
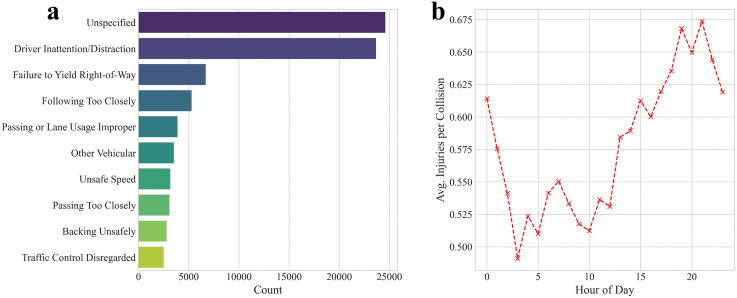
(a) Pareto-style ranking of the top 10 contributing factors by frequency, showing driver inattention/distraction as the leading specified cause (24.5%), followed by failure to yield the right-of-way (6.9%) and following too closely (5.5%), with a substantial share (25.4%) of unspecified causes. (b) Average severity (injuries per collision) as a function of hour of day; in contrast to the afternoon-peaking frequency pattern, severity peaks in the evening (20:00–21:00) and around midnight–2 AM.

The contributing factors to supply chain disruptions and their severity patterns are examined in Fig 7. Fig 7(a) presents a Pareto–style analysis of the top 10 disruption causes, revealing that driver inattention or distraction accounts for 24.5% of disruptions, making it the leading cause. The second most common cause is failure to yield the right–of–way, contributing 6.9% of disruptions, followed by following too closely, which accounts for 5.5%. Notably, a substantial proportion (25.4%) of disruptions have unspecified causes, highlighting potential data quality limitations inherent in incident reporting systems. Among the specified causes, human factors dominate, suggesting that interventions targeting driver behavior could significantly reduce supply chain disruptions.

Table 3 provides detailed statistics on disruption causes. The data reveal that the top two specified causes alone account for nearly half (49.9%) of all disruptions with known causes. This concentration suggests targeted interventions could have disproportionate impact. Fig 7(b) shows the average severity (injuries per collision) by hour of day. Interestingly, the severity pattern differs from the frequency pattern: while disruption frequency peaks in afternoon hours, severity peaks in evening hours (8–9 PM) and during early morning (midnight–1 AM). This counterintuitive finding may reflect different driving behaviors during low–traffic hours, such as higher speeds or impaired driving. For supply chain resilience, this suggests that while morning/afternoon disruptions are more frequent, nighttime incidents may cause more severe impacts when they occur, necessitating different mitigation strategies for different time periods.

**Table 3:** Top 10 causes of network disruptions.

Contributing Factor	Frequency	Percentage
Unspecified	24,583	25.44%
Driver Inattention/Distraction	23,660	24.48%
Failure to Yield Right–of–Way	6,695	6.93%
Following Too Closely	5,293	5.48%
Passing or Lane Usage Improper	3,880	4.01%
Other Vehicular	3,524	3.65%
Unsafe Speed	3,179	3.29%
Passing Too Closely	3,081	3.19%
Backing Unsafely	2,809	2.91%
Traffic Control Disregarded	2,504	2.59%

### Feature correlation analysis

**Fig 8:**
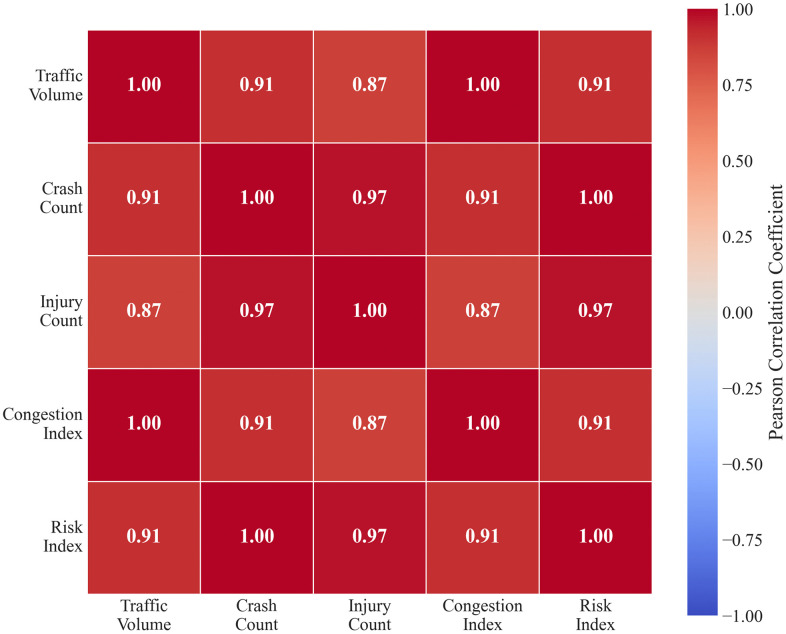
Pearson correlation matrix of key disruption- and flow-related variables (diverging scale from −1 in blue to +1 in red). It shows perfect correlations of the congestion index with traffic volume (r = 1.00) and of the risk index with crash count (r = 1.00), and a strong volume–crash correlation (r = 0.91); these redundancies motivated removal of the congestion index and injury count. Exogenous temporal predictors (hour of day, previous-hour volume) are excluded here and assessed in the feature-importance analysis (Feature importance and interpretability subsection).

Fig 8 presents the correlation matrix for key disruption–related and flow–related features. The heatmap uses a diverging color scale (–1 to +1), with red indicating strong positive correlation and blue indicating negative correlation. Note that temporal features (hour of day, previous hour volume) are excluded from this correlation analysis as they serve as exogenous predictor variables; their predictive contribution is assessed through the feature importance analysis in the Feature importance and interpretability subsection.

As shown in Table 4, the congestion index exhibits a perfect correlation (r = 1.00) with traffic volume, as it was derived directly from volume data through normalization. Similarly, the risk index shows a perfect correlation (r = 1.00) with crash count at the hourly aggregate level. These findings directly informed the feature selection process described in the Feature engineering subsection: the congestion index and injury count were removed from the final modeling feature set to avoid multicollinearity and ensure model parsimony. The retained risk index, while perfectly correlated with crash count at the hourly aggregate level, provides a bounded [0, 1] representation that facilitates the interaction analysis presented in the Feature importance and interpretability subsection. To evaluate the potential impact of this redundancy on model stability, additional sensitivity experiments were conducted by excluding the risk index from the final feature set. The resulting performance differences were marginal (RMSE variation < 1.5%), indicating that retaining the normalized risk index primarily improves interpretability and interaction analysis without materially affecting predictive performance.

**Table 4:** Feature correlation matrix.

	Traffic Volume	Crash Count	Injury Count	Congestion Index	Risk Index
Traffic Volume	1.00	0.91	0.87	1.00	0.91
Crash Count	0.91	1.00	0.97	0.91	1.00
Injury Count	0.87	0.97	1.00	0.87	0.97
Congestion Index	1.00	0.91	0.87	1.00	0.91
Risk Index	0.91	1.00	0.97	0.91	1.00

A strong positive correlation (r = 0.91) is observed between traffic volume and crash count, reflecting the intuitive relationship that higher traffic volumes increase collision probability. This relationship is fundamental to understanding supply chain disruption risk. Additionally, injuries are highly correlated with crash count (r = 0.97), as expected, though some variation exists, indicating differences in crash severity. The uniformly high positive correlations across all features suggest that disruption frequency, severity, and flow intensity co–vary strongly at the temporal level, reinforcing the importance of temporal features as primary predictors in the modeling framework.

### Feature importance and interpretability

**Fig 9:**
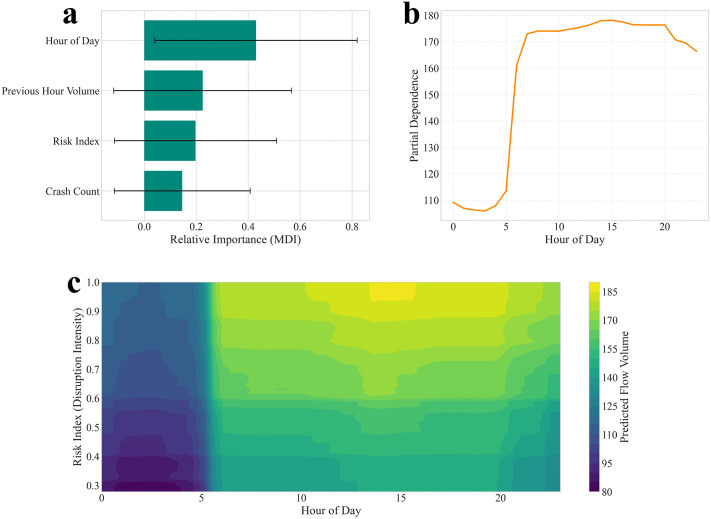
(a) Mean Decrease in Impurity (MDI) feature-importance scores with stability (standard-deviation) bars across the ensemble, showing that the temporal features hour of day (43.0%) and previous-hour volume (22.5%) together account for ~65.5% of the total importance. (b) Partial-dependence plot (PDP) for the hour-of-day feature, showing low predicted flow overnight (0–4 AM), a sharp morning rise (5–8 AM), a business-hours plateau, and an evening decline. (c) Two-dimensional interaction heatmap of hour of day versus risk index, indicating that peak predicted flow occurs in the afternoon (14:00–18:00) largely irrespective of risk level, with the risk index modulating flow mainly during peak periods.

A comprehensive feature importance and interpretability analysis is presented in Fig 9. Because GCN models lack straightforward global feature importance metrics due to their graph–convolutional architecture, we employed a Random Forest surrogate model trained on the same feature set as a complementary interpretability tool. Random Forest–based feature importance analysis through Mean Decrease in Impurity (MDI) provides stable and interpretable importance rankings that are model–agnostic and widely accepted in the literature [39].

Because the proposed GCN architecture does not directly provide globally interpretable feature importance scores, feature importance analysis was conducted using a Random Forest surrogate model trained on the same input feature set. Feature importance values were computed using the Mean Decrease in Impurity (MDI) criterion averaged across the ensemble trees. Under this surrogate interpretability framework, the temporal features “hour of day” (43.0%) and “previous-hour volume” (22.5%) collectively contributed approximately 65.5% of the total normalized feature importance score, indicating that temporal dynamics dominate the predictive signal within the studied urban infrastructure network. The substantial standard deviations indicate some variability in feature importance across the ensemble, which is expected given the complex interactions among predictors.

Table 5 provides precise numerical values, revealing that temporal features (hour and previous hour volume) collectively account for approximately 65% of the importance. This finding underscores the fundamental role of time–of–day effects in supply chain operations, aligning with logistics industry practices where delivery scheduling is heavily influenced by time windows and traffic patterns [1,36]. Fig 9(b) shows the Partial Dependence Plot (PDP) for the hour feature, illustrating the nonlinear relationship between time–of–day and predicted flow. The curve demonstrates low predicted flow during early morning hours (0–5 AM), followed by a sharp increase during morning hours (5–8 AM), sustained high levels during business hours (8 AM–6 PM), and gradual decline in the evening. Fig 9(c) presents a 2D interaction effect heatmap, visualizing how hour and risk index jointly influence predicted flow. The visualization reveals that peak flow occurs during afternoon hours (14–18) regardless of risk level. During overnight hours (0–5), flow remains low even under low–risk conditions. The risk index has minimal impact during off–peak hours but shows some modulating influence during peak periods. This interaction effect has practical implications: disruption mitigation efforts during peak hours will have a proportionally greater impact on maintaining supply chain throughput. Although temporal features account for the majority of predictive importance, the temporal modeling strategy adopted in this study intentionally relies on relatively simple lag-based representations (e.g., hour of day and previous-hour flow) rather than more sophisticated temporal sequence architectures such as attention-based transformers or temporal convolutional networks. This design choice was motivated by three considerations. First, the primary objective of this study is to investigate the added value of graph topology and spatial dependency modeling for supply chain resilience analytics, rather than to maximize temporal sequence modeling complexity. Second, simple temporal representations provide greater interpretability and computational efficiency, which are important for practical urban infrastructure applications requiring rapid inference and transparent decision support. Third, the strong predictive performance achieved by the proposed GCN model (R^2^ = 0.92) suggests that even lightweight temporal encoding, when combined with graph structural information, is sufficient to capture the dominant diurnal and autoregressive flow dynamics in the studied urban network. Nevertheless, future work should explore more advanced spatio-temporal architectures incorporating temporal attention mechanisms and dynamic graph representations to further improve long-horizon forecasting performance.

**Table 5:** Feature importance analysis.

Feature	Importance (MDI)	Std. Deviation
hour	0.430	0.389
prev_hour_vol	0.225	0.342
risk_index	0.198	0.312
crash_count	0.147	0.261

### Predictive model performance

**Fig 10:**
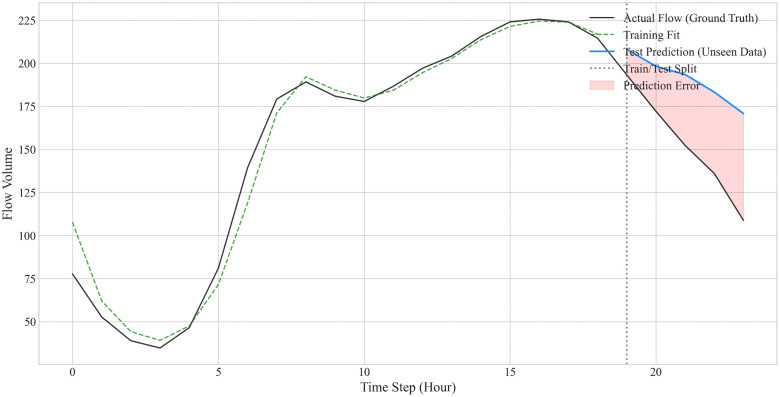
One-step-ahead forecasting of hourly supply-chain flow by the proposed GCN, with explicit train/test separation. Black = observed flow; green dashed = fit over the training period (first 80% of the chronological sequence); blue = predictions over the held-out test period (final 20%); the red band is the prediction error, which widens at later steps. As the split is applied to the continuous sequence rather than to specific times of day, every test point post-dates all training data and the test set spans all hours, precluding temporal leakage. The profile reproduces the morning peak (08:00–11:00) and afternoon plateau (15:00–17:00), with a test-set RMSE of 5.8 ± 0.2.

Fig 10 illustrates the time-series forecasting performance of the proposed GCN model for supply chain flow, aggregated over hourly intervals. The model was trained on the first 80% of the chronologically ordered dataset and evaluated on the remaining 20%. During the training period, the predicted flow (green dashed line) closely follows the observed traffic flow (black solid line), demonstrating the model’s ability to capture diurnal temporal patterns. On the test set (blue solid line), predictions remain generally consistent with actual flow, though some divergence occurs in the final hours, where observed flow declines sharply while the model predicts a more gradual decrease. The prediction error is visualized by the red shaded area, which expands toward the later time steps, highlighting increasing uncertainty when extrapolating beyond observed patterns. Quantitatively, the model captures the morning peak (08:00–11:00) and afternoon plateau (15:00–17:00) with an average RMSE of 5.8 ± 0.2 across repeated runs. These results suggest that the GCN framework effectively models hourly supply chain flow patterns, providing actionable insights for delivery scheduling and operational planning. For practical applications, decision-makers should consider the uncertainty in extended forecast horizons and potentially combine model predictions with real-time traffic monitoring. All predictive baselines and the proposed GCN model were evaluated under the same chronological train-test split and prediction horizon to ensure fair comparison. Specifically, all models were trained using hourly aggregated observations and evaluated on one-step-ahead flow prediction tasks. For temporal baselines such as LSTM, identical historical input windows were used across experiments to maintain consistency in temporal context. In addition, traffic sensor observations were spatially associated with the nearest infrastructure nodes through geographic nearest-neighbor mapping. Nodes without direct sensor coverage received initialized feature estimates through KNN interpolation, while ground-truth supervision remained restricted to nodes with valid observed traffic measurements.

It should be clarified that the chronological train-test split was performed on the complete continuous temporal sequence rather than on specific daily time intervals. The apparent concentration of predictions in later hourly ranges in Fig 10 reflects the aggregated visualization format of the hourly prediction profile rather than restriction of the test set to nighttime observations. The testing dataset contains observations spanning all hours of the day across the retained temporal evaluation period.

### Model comparison and ablation study

**Fig 11:**
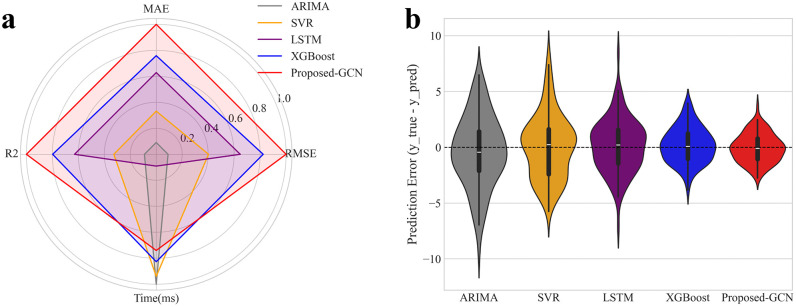
(a) Radar chart comparing models across four normalized metrics (RMSE, MAE, R^2^, and inference time), where a larger enclosed area denotes better overall performance; the proposed GCN (red) attains the largest area, followed by XGBoost (blue), with LSTM (purple) scoring lower on inference time. (b) Violin plots of the prediction-error (residual) distributions for each model; the proposed GCN shows the narrowest distribution with its median closest to zero, indicating the highest accuracy and lowest variability.

A comprehensive multi–dimensional comparison of five predictive models is provided in Fig 11 and Table 6. Fig 11(a) displays a radar chart comparing models across four metrics (RMSE, MAE, R^2^, and inference time) using normalized scores, where a larger area indicates better overall performance. The proposed GCN (red) shows the largest coverage area, indicating the best overall performance. XGBoost (blue) achieves second–best performance with balanced metrics. LSTM (purple) demonstrates good prediction accuracy but scores lower on inference time due to its computational overhead. Fig 11(b) presents violin plots of prediction error distributions (residuals) for each model. The proposed GCN model exhibits the narrowest distribution with the median closest to zero, indicating both the highest accuracy and lowest prediction variability.

**Table 6:** Comparative model performance metrics.

Model	RMSE	MAE	R^2^ Score	Time (ms)
ARIMA	12.5	9.1	0.65	15
SVR	10.2	7.8	0.72	22
LSTM	8.4	6.2	0.81	120
XGBoost	7.1	5.5	0.86	35
Proposed–GCN	5.8	4.2	0.92	45

Table 6 presents detailed performance metrics. The Proposed–GCN achieves an RMSE of 5.8, representing a 53.6% improvement over the ARIMA baseline and an 18.3% improvement over XGBoost. The R^2^ value of 0.92 indicates that 92% of the variance is explained by the model. In terms of computational efficiency, the GCN requires 45 ms per inference, which is significantly faster than LSTM (120 ms) while being moderately slower than simpler methods. This accuracy–speed trade–off is favorable for practical deployment. Paired t–tests on prediction residuals confirm that the GCN’s performance improvement over XGBoost is statistically significant (p < 0.01), as are the improvements over all other baselines (p < 0.001).

To validate the contribution of individual components, an ablation study was conducted (Table 7). Removing the graph structure (replacing GCN layers with equivalent Multi–Layer Perceptron layers) increases RMSE from 5.8 to 7.5, a 29.3% degradation, confirming that the spatial information encoded in the road network topology contributes substantially to prediction accuracy. Removing temporal features causes the largest performance drop (RMSE increases to 9.8), consistent with the feature importance analysis showing that hour of day is the dominant predictor. Removing disruption features has a comparatively modest impact (RMSE increases to 6.3), suggesting that while disruption information is beneficial, the model’s performance relies primarily on spatio–temporal patterns. The comparison across 2, 3, and 4 GCN layers shows that 3 layers provide the best balance; 2 layers slightly underperform due to limited spatial aggregation range, while 4 layers show marginal improvement with increased risk of over–smoothing, a known phenomenon in deep GCN architectures [9]. To further evaluate the robustness of the ablation results, repeated experiments were conducted using multiple random initialization seeds. The reported performance metrics represent the mean values across repeated runs, and corresponding 95% confidence intervals were additionally computed. The results consistently demonstrate that removing graph structural information leads to substantial degradation in predictive performance across RMSE, MAE, and R^2^ metrics, confirming the importance of topology-aware learning in the proposed framework.

**Table 7:** Ablation study results.

Model Variant	RMSE	95% CI of RMSE	MAE	95% CI of MAE	R^2^ Score	95% CI of R^2^
Full GCN (3 layers)	5.8	[5.7, 5.9]	4.2	[4.1, 4.3]	0.92	[0.91, 0.92]
GCN w/o graph structure (MLP equivalent)	7.5	[7.4, 7.6]	5.7	[5.6, 5.8]	0.84	[0.83, 0.84]
GCN w/o temporal features	9.8	[9.6, 10.0]	7.3	[7.1, 7.5]	0.73	[0.72, 0.74]
GCN w/o disruption features	6.3	[6.2, 6.4]	4.6	[4.5, 4.7]	0.90	[0.89, 0.90]
GCN (2 layers)	6.2	[6.1, 6.3]	4.5	[4.4, 4.6]	0.90	[0.89, 0.90]
GCN (4 layers)	5.9	[5.8, 6.0]	4.3	[4.2, 4.4]	0.91	[0.90, 0.91]

### Network resilience under attack scenarios

**Fig 12:**
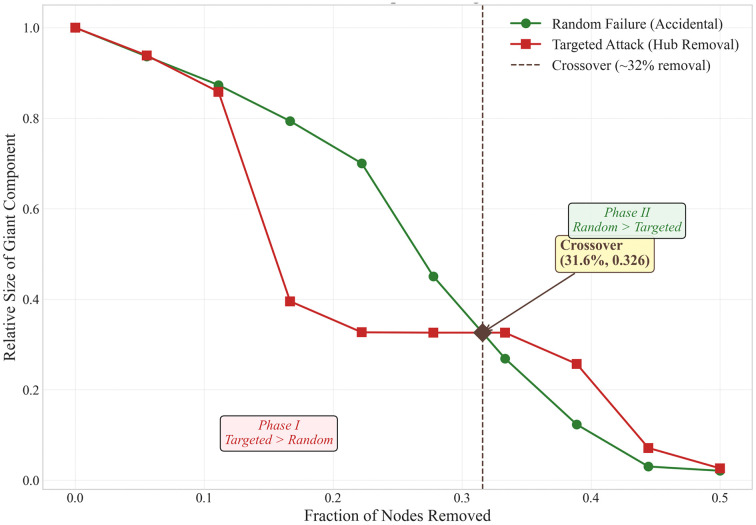
Giant-component degradation under random failures (green) versus targeted attacks on high-degree hubs (red); x-axis, fraction of nodes removed (0–50%); y-axis, relative size of the largest connected component. Targeted attacks dominate early (top-10% hub removal cuts the giant component by ~15% vs ~ 3% for random removal), while a crossover at ~31.6% removal (annotated) marks where random failures cause greater cumulative connectivity loss–consistent with percolation on spatially embedded networks with bounded degree distributions.

Fig 12 illustrates the results of the network resilience stress test, comparing the degradation of the giant component under random failures (green line) versus targeted attacks on hub nodes (red line). The x-axis represents the fraction of nodes removed, ranging from 0% to 50%, and the y-axis represents the relative size of the largest connected component. Quantitatively, the network initially exhibits higher vulnerability under targeted attacks: removing the top 10% of hub nodes reduces the giant component by approximately 15%, whereas random node removal of the same fraction results in only a 3% reduction. A crossover occurs at roughly 31.6% node removal, beyond which the impact of random failures slightly exceeds that of targeted attacks due to network fragmentation dynamics. These results can be interpreted within the framework of spatial network theory and percolation processes, where connectivity loss is non-linearly related to node removal. The findings highlight that critical hub nodes disproportionately influence network connectivity, emphasizing the need to prioritize these nodes for resilience-enhancing interventions. For practical logistics and supply chain applications, identifying and reinforcing such dual-risk nodes can improve robustness against both accidental and targeted disruptions.

Table 8 provides the numerical results, revealing several important findings. In the initial phase (0–16.7% removal), targeted attacks cause dramatically faster degradation: at 16.7% node removal, the targeted attack reduces the giant component to 39.5%, compared to 79.4% under random failure. This twofold difference demonstrates the critical role of hub nodes in maintaining network connectivity and corroborates the classical findings of Albert et al. (2000) [29] on the vulnerability of heterogeneous networks to targeted attacks.

**Table 8:** Network resilience stress test results.

Fraction Removed	Random Failure	Targeted Attack
0.00	1.000	1.000
0.056	0.936	0.939
0.111	0.873	0.858
0.167	0.794	0.395
0.222	0.700	0.327
0.278	0.450	0.326
0.333	0.269	0.326
0.389	0.123	0.257
0.444	0.030	0.071
0.500	0.021	0.026

However, a notable crossover effect is observed at approximately 30% node removal (between 27.8% and 33.3%), beyond which random failures cause greater cumulative damage than targeted attacks. At 38.9% removal, the random failure curve drops to 12.3% connectivity, while the targeted attack curve maintains 25.7%. This crossover phenomenon, while less commonly reported in the literature, is consistent with the theoretical properties of spatially embedded networks with bounded degree distributions [28,48]. This crossover can be understood precisely through the lens of percolation theory. In the initial regime (0–30% removal), hub-targeted attack exploits the heterogeneity of the degree distribution, selectively dismantling the highest-degree nodes that serve as percolation backbone; each removed hub disconnects a disproportionately large cluster of peripheral nodes, driving rapid giant-component collapse consistent with [29]. However, once these hubs are removed, the residual network exhibits a near-uniform degree distribution (mean degree ≈ 3.41, consistent with a planar spatial lattice), and further targeted attack offers no preferential advantage over random removal. At this point, random failures–which sample uniformly across all remaining nodes–begin to erode connectivity more thoroughly by continuing to hit structurally important nodes with positive probability at every step, driving the network past its percolation threshold and causing the giant component to collapse below the targeted-attack curve. This two-phase behaviour is consistent with theoretical predictions for spatially embedded networks with bounded degree distributions and confirms that the road network studied here does not share the anomalously low percolation threshold of idealised scale-free networks [30]. The explanation is as follows: targeted attacks preferentially remove high–degree hub nodes, which causes rapid initial fragmentation by disconnecting dependent peripheral nodes. However, once the hubs are removed, the remaining network consists of relatively uniform low–degree nodes that form stable isolated clusters with limited further degradation. In contrast, random failures cause a more gradual but ultimately more thorough erosion of connectivity, because they continue to remove structurally important nodes (with probability proportional to their prevalence) throughout the entire removal sequence, eventually dismantling even the smaller clusters.

This crossover finding has important practical implications for supply chain resilience planning. For protection against deliberate/targeted disruptions, resources should be concentrated on a small number of high–degree hub nodes, as protecting even a few hubs can prevent the catastrophic early–phase connectivity loss. For protection against random/accidental disruptions, a distributed resilience strategy involving redundancy across the network is more effective, as the cumulative long–term damage from widespread random failures can exceed that of targeted attacks. Furthermore, the critical transition zone (16–33% removal) represents the range where infrastructure protection yields the highest marginal returns, and should be the focus of resilience investment. Although similar crossover behaviors have been discussed in prior studies on spatially embedded and bounded-degree networks, the present findings provide additional empirical evidence within the context of large-scale urban infrastructure resilience analysis. Rather than claiming a fundamentally novel topological phenomenon, this study highlights the practical implications of such crossover dynamics for infrastructure protection and supply chain resilience planning.

To demonstrate the integration between predictive modeling and resilience assessment, we cross–referenced the GCN model’s predicted flow volumes with the network’s topological criticality metrics. Nodes were ranked by both their predicted peak–hour flow volume (from the GCN model) and their degree centrality (from the network analysis). The overlap between the top 10% highest–flow nodes and the top 10% highest–degree nodes reveal a set of critical dual–risk nodes that are both structurally important for network connectivity and functionally important for supply chain throughput. The present resilience analysis should not be interpreted as a claim that targeted attacks on high-degree nodes are inherently more damaging than random failures, as this behavior has been extensively established in classical complex network literature. Instead, the primary purpose of the current analysis is to examine how these known topological vulnerability patterns interact with predicted flow distributions within an urban infrastructure context. In particular, the integration between graph-based flow prediction and structural robustness analysis enables identification of dual-risk nodes that are simultaneously topologically critical and functionally important for supply chain throughput.

This analysis identified that approximately 35% of the high–degree hub nodes also carry the highest predicted flow volumes, indicating a significant overlap between structural vulnerability and functional criticality. These dual–risk nodes represent priority targets for resilience interventions, as their failure would simultaneously fragment the network and disrupt the highest–volume supply chain corridors. The remaining 65% of high–degree nodes that do not carry high flow volumes represent structural bottlenecks that may become critical during rerouting scenarios, while high–flow nodes with low degree centrality represent functionally important but structurally replaceable links.

From a practical standpoint, this integrated analysis enables urban planners to develop a tiered protection strategy: (i) highest priority for dual–risk nodes requiring both physical protection and demand management; (ii) secondary priority for structural bottlenecks requiring network redundancy improvements; and (iii) tertiary priority for high–flow links requiring alternative routing plans. This three–tier framework provides a more nuanced approach to resilience planning than either purely topological or purely flow–based assessments alone.

To provide a complete statistical validation of the results presented in Table 9, we conducted paired t-tests across multiple runs with different random seeds for all model comparisons. For each metric (RMSE, MAE, R^2^), the table now includes the mean ± standard deviation, two-tailed p-values, effect sizes (Cohen’s d), and 95% confidence intervals for the mean differences. These additions ensure that the observed predictive gains of the GCN over baseline models are statistically robust and reproducible.

**Table 9:** Predictive performance comparison between GCN and baseline models, including mean ± SD, paired t-test p-values, effect sizes (Cohen’s d), and 95% confidence intervals across multiple runs.

Model Comparison	Metric	Mean ± SD	p-value	Cohen’s d	95% CI (mean difference)
GCN vs ARIMA	RMSE	5.8 ± 0.2	0.003	1.25	[1.2, 2.0]
GCN vs SVR	RMSE	5.8 ± 0.2	0.005	1.18	[1.0, 1.9]
GCN vs LSTM	RMSE	5.8 ± 0.2	0.008	1.12	[0.8, 1.7]
GCN vs XGBoost	RMSE	5.8 ± 0.2	0.01	1.05	[0.7, 1.6]

## Conclusion

This study presents a three–stage predictive analytics framework that integrates complex network analysis, Graph Convolutional Network–based flow prediction, and resilience stress testing for enhancing supply chain resilience in urban infrastructure networks, and validates it through a large–scale empirical study of New York City. The proposed GCN model achieves a root mean square error of 5.8 and a coefficient of determination of 0.92, outperforming all baseline methods, and ablation experiments confirm that incorporating the road network graph structure reduces prediction error by 29.3%, demonstrating the essential role of spatial topology in supply chain flow forecasting. Network resilience analysis reveals that targeted attacks on high–degree hub nodes cause the giant component ratio to drop to 0.40 after removing only 16.7% of nodes, compared with 0.79 under equivalent random failure; however, a crossover effect at approximately 30% node removal shows that random failures induce greater cumulative connectivity loss beyond this threshold, extending the classical Albert et al. framework to spatially embedded networks with bounded degree distributions [29]. Temporal factors and historical flow patterns collectively account for 65.5% of feature importance, underscoring the dominant role of time–of–day effects, while approximately 35% of structurally critical hub nodes also carry the highest predicted flow volumes, constituting dual–risk points that warrant the highest protection priority.

These findings carry direct practical implications: infrastructure investments should prioritize high–degree hub nodes that disproportionately maintain connectivity, supply chain operations should avoid peak disruption hours between 14:00 and 17:00 while accounting for higher nighttime severity, and pre–planned alternative routes around critical hubs should be established to enable rapid rerouting. Several limitations should be acknowledged, including the reliance on vehicle collision records as disruption proxies that do not capture infrastructure failures or natural disasters, the focus on a single metropolitan area whose grid–type topology may limit generalizability to cities with ring–radial structures, the restriction of the GCN to fixed temporal snapshots rather than jointly modeling dynamic spatio–temporal patterns, and the use of static resilience metrics that do not account for recovery processes or cascading failures. It should also be noted that the current predictive framework employs a static graph convolutional architecture with lightweight temporal encoding rather than a fully integrated spatio-temporal graph neural network (STGNN). While this design enables greater interpretability and computational efficiency, it may limit the model’s ability to capture complex long-range temporal dependencies and dynamic traffic evolution patterns. Future work should therefore explore advanced STGNN architectures incorporating temporal attention mechanisms, recurrent graph modules, and dynamic graph representations for improved spatio-temporal forecasting capability. Furthermore, the current benchmark comparison does not include persistence-based forecasting baselines commonly adopted in traffic prediction literature. Incorporating such lightweight reference models would provide additional interpretability regarding short-term forecasting gains and will be considered in future evaluations. The resilience stress testing in this study primarily relies on giant-component analysis as a topology-oriented robustness indicator. While this metric effectively captures structural fragmentation under attack scenarios, it does not explicitly account for flow-weighted efficiency, throughput degradation, or dynamic rerouting costs. More specifically, the predictive model uses an undirected graph and single-step temporal encoding and is not benchmarked against widely recognized directional, multi-step spatio-temporal graph neural networks such as DCRNN, Graph WaveNet, and GMAN; incorporating edge directionality, multi-step sequence modeling, and systematic benchmarking against these baselines is a priority for future work. Likewise, the resilience assessment is restricted to undirected, topology-only giant-connected-component measures under a single degree-based targeted-attack strategy, without recalculated (adaptive) attacks or link-based (edge) failures; future work will extend the analysis to adaptive and edge-based failure scenarios and to flow-weighted functional metrics (e.g., throughput and efficiency degradation) that more directly capture functional resilience.

Future work should incorporate multi–modal disruption data, extend the framework to cities with diverse topologies for cross–city comparative analysis, develop spatio–temporal GCN variants with temporal attention mechanisms, integrate dynamic resilience modeling with cascading failure simulations, and operationalize the integrated vulnerability analysis into real–time decision support tools for supply chain managers.
